# “If I’d Had Something Like SAFE at the Time, Maybe I Would’ve Left Him Sooner.”—Essential Features of eHealth Interventions for Women Exposed to Intimate Partner Violence: A Qualitative Study

**DOI:** 10.1177/08862605211036108

**Published:** 2021-08-06

**Authors:** Nicole van Gelder, Suzanne Ligthart, Julia ten Elzen, Judith Prins, Karin van Rosmalen-Nooijens, Sabine Oertelt-Prigione

**Affiliations:** 1 Radboud University Medical Center, Nijmegen, Netherlands

**Keywords:** intimate partner violence abuse, domestic violence, women, eHealth, interview, qualitative, survivors

## Abstract

Approximately one in three women worldwide experiences intimate partner violence and abuse (IPVA) in her lifetime. Despite its frequent occurrence and severe consequences, women often refrain from seeking help. eHealth has the potential to remove some of the barriers women face in help seeking and disclosing. To guarantee the client-centeredness of an (online) intervention it is important to involve the target group and people with expertise in the development process. Therefore, we conducted an interview study with survivors and professionals, in order to assess needs, obstacles, and wishes with regard to an eHealth intervention for women experiencing IPVA. Semi-structured interviews were conducted with 16 women (8 survivors and 8 professionals) between 22 and 52 years old, with varied experiences of IPVA and help. Qualitative data was analyzed using a grounded theory approach and open thematic coding. During analysis we identified a third stakeholder group within the study population: survivor-professionals, with both personal experiences of and professional knowledge on IPVA. All stakeholder groups largely agree on the priorities for an eHealth intervention: safety, acknowledgment, contact with fellow survivors, and help. Nevertheless, the groups offer different perspectives, with the survivor-professionals functioning as a bridge group between the survivors and professionals. The groups prioritize different topics. For example, survivors and survivor-professionals highlighted the essential need for safety, while professionals underlined the importance of acknowledgment. Survivor-professionals were the only ones to emphasize the importance of addressing various life domains. The experiences of professionals and survivors highlight a broad range of needs and potential obstacles for eHealth interventions. Consideration of these findings could improve the client-centeredness of existing and future (online) interventions for women experiencing IPVA.

## Abbreviations

COREQ = COnsolidated criteria for REporting Qualitative research, DV = domestic violence, GCQ = General Characteristics Questionnaire, GDPR (*AVG in Dutch*) = General Data Protection Regulation, GREVIO = Group of Experts on Action against Violence against Women and Domestic Violence, IPVA = intimate partner violence and abuse, PTSD = post-traumatic stress disorder, RCT = randomized controlled trial, WHO = World Health Organization.

## Background

The World Health Organization defines intimate partner violence and abuse (IPVA) as any physical, sexual, psychological, or economic violence that occurs between former or current partners (WHO, 2013). While various terminology is used in research to describe IPVA, such as domestic violence (DV), partner abuse, abused women, and abusive relationships, we choose to consistently use the term IPVA. Both men and women can experience IPVA; however, women are more frequently affected by it (Tjaden & Thoennes, 2000). Worldwide approximately one in three women experiences at least one type of IPVA in her lifetime (FRA, 2014; WHO, 2021). In a survey conducted in the Netherlands in 2019 6.2% of Dutch adult women reported physical and/or sexual IPVA in the last five years (Ten Boom & Wittebrood, 2019). Furthermore, almost 60% of all femicides in the Netherlands between 2015 and 2019 were committed by an (ex-)partner (Centraal Bureau voor de Statistiek, 2020a).

IPVA has negative consequences at various levels: physical (e.g., injuries), mental (e.g., anxiety, depression, post-traumatic stress disorder [PTSD]), social (e.g., distrust, social isolation), professional, and financial (e.g., job loss). Growing up in a violent household also impacts the lives of children, who can witness abuse or be directly exposed to it. Childhood exposure to IPVA increases the risk of becoming a perpetrator and/or victim of IPVA later in life due to intergenerational transmission (Ehrensaft et al., 2003; Ellsberg et al., 2008; Garcia-Moreno et al., 2006).

Despite its frequent occurrence and its severe consequences, women often refrain from seeking help and disclosing the violence. The possible explanations for this hesitance are fear, shame, guilt, (social) isolation, love, hope that the partner will change, distrust in professional help, worries about the children, financial worries, unawareness of IPVA, and lack of knowledge about support options (Hegarty & Taft, 2001; O’Doherty et al., 2016; Petersen et al., 2005; Rodríguez et al., 2009; Wilson et al., 2007). Not all women have the opportunity to physically reach out for help or visit a supporting organization. Internet and mobile solutions represent an option to address these barriers. An internet-based or eHealth intervention is available at all times, it is easily accessible from various devices, and can offer the benefit of anonymity. It can be especially helpful for women who are unsure about whether they are dealing with IPVA or who are contemplating seeking help. It has the opportunity to bring together various aspects of supporting survivors such as information, help options, and support from professionals and fellow survivors, in a low threshold manner. This can help survivors in reflecting on their own situation, in help seeking, and in feeling supported while providing anonymity, privacy, and autonomy. However, we have to take into account that not everyone has access to the internet, online means have a limited ability in assessing a survivor’s situation and possible danger, and an abusive partner may discover the online help seeking actions if they keep an eye on the survivor’s online presence.

The development of eHealth interventions for women exposed to IPVA is a novel field of practice-oriented research, which has yielded positive results in the USA, New Zealand, Australia, and Canada (Table 1). There are no scientifically developed and evaluated eHealth interventions for the European area this far, despite survivors and professionals being supportive of using eHealth (for IPVA) (Mantler et al., 2018; Tarzia et al., 2017; Verhoeks, Teunissen et al., 2017; White et al., 2016). Currently, most people in the Netherlands have access to the internet and are digitally literate, which facilitates the use of eHealth of the people aged 12 years and older, 97% has access to the internet and 92.1% has a smartphone or mobile phone (Centraal Bureau voor de Statistiek, 2020b, 2020c). Lastly, GREVIO (Group of Experts on Action against Violence against Women and Domestic Violence) “encourages the efforts made [in the Netherlands] to carry out research to determine whether provision of information via digital means is effective.” (GREVIO, 2020, p. 32).

**Table 1.** Outcomes of Online Interventions for Women Exposed to IPVA.

Authors	Intervention	Outcomes
(Glass et al., 2010)	Computerized safety decision aid (USA)	Evaluation study: The intervention decreased decisional conflict and increased feelings of support in the safety planning process.
(Constantino et al., 2015)	HELPP (USA)	RCT: HELPP decreased anxiety, depression, and anger. It increased personal and social support. HELPP online proved to be more effective than HELPP face-to-face.
(Eden et al., 2015)	IRIS (USA)	RCT: IRIS decreased uncertainty, feeling unsupported, and decisional conflict with regard to personal safety, more so than for the control group.
(Koziol-McLain et al., 2018)	iSAFE (NZL)	RCT: iSAFE reduced violence and symptoms of depression for Maori-women. Non-Maori women did not experience this. Both the intervention and control group found iSAFE useful.
(Hegarty et al., 2019)	I-DECIDE (AUS)	RCT: No difference was found between the intervention and control group. Women in both groups reported increased self-efficacy and decreases in depression and fear of partner. Process evaluation: Both groups experienced increased awareness, self-efficacy, and perceived support.
(Ford-Gilboe et al., 2020)	iCan Plan 4 Safety (CAN)	RCT: Women in the intervention and control group experienced decreases in depression, PTSD, coercive control, and decisional conflict. They experienced increases in helpfulness of safety actions, confidence in safety planning, social support, and mastery (control over own life). Process evaluation: All participants reported high levels of benefit, safety, accessibility of the interventions, and low risk of harm. Women were more positive about helpfulness and fit when they received a tailored intervention.

*Note.* RCT = randomized controlled trial, PTSD = post-traumatic stress disorder.

It is crucial to address the needs and wishes of the target group while developing an intervention. Evaluations from IPVA eHealth interventions show that while survivors feel online support cannot substitute offline support, they found it useful and they value the advantages that eHealth can provide, such as accessibility, privacy, autonomy, no judgment, and feeling supported (Ford-Gilboe et al., 2020; Hegarty et al., 2019; Lindsay et al., 2013; Tarzia et al., 2017). They also express concerns with regard to the possible consequences when an abusive partner discovers the efforts to seek help online (Lindsay et al., 2013). Little studies have assessed professionals’ views in a similar way for IPVA eHealth interventions. Professionals from women’s shelters state that technology can help with reaching more women, creating more possibilities for communication, and accessibility (Mantler et al., 2018). In studies assessing (mental) health professionals’ attitudes toward eHealth, professionals believe that eHealth can be beneficial for their patients in treatment outcomes, communication, and accessibility. However, limited access to the internet for certain people is an obstacle (White et al., 2016). Different stakeholders can offer different perspectives in the process of developing an eHealth intervention for IPVA survivors. Survivors can speak from their own experience and provide first-hand information on how to best approach the target group, what to provide to them, and what to take into consideration when working with survivors of IPVA. Professionals working with survivors can offer information on logistics, options, and constraints inherent to the support process. Given their distinct but overlapping points of view, we asked both survivors and professionals to share their expertise with us to aid the development of the first eHealth platform for women experiencing IPVA in Europe.

### Interview Study and SAFE

This interview study focused on needs and wishes of survivors and professionals regarding online help. This data, together with elements from international eHealth interventions and the scientific literature, was used toward the development of “SAFE: an eHealth intervention for women exposed to IPVA in the Netherlands.” The previously published SAFE protocol describes the intervention, RCT, process evaluation and open feasibility study (van Gelder et al., 2020). The following is the research question for this interview study: *Which key aspects should be addressed in the development of SAFE, an eHealth intervention for women exposed to IPVA, according to survivors and professionals?*

## Methods

### Study Design and Data Acquisition

We used the COnsolidated criteria for REporting Qualitative research (COREQ) as a guideline for reporting this study (Tong et al., 2007). In this qualitative study, we used grounded theory to investigate the needs of women exposed to IPVA (Henning et al., 2004). The study entails 16 semi-structured interviews with women who experienced IPVA and professionals in the field of DV/IPVA. One researcher (NvG) conducted interviews until saturation was reached. NvG is trained in psychological conversational skills as a pedagogue and trained specifically for these interviews by KvRN, who conducted interviews with adolescents regarding DV (van Rosmalen-Nooijens et al., 2017). A flexible interview guide was used (Supplemental Material). After every four or five interviews, the participants’ answers and the interview guide were evaluated. As data saturation took place in the interview process, we added new subquestions when needed. Before the start of the interview the participant received an information letter and signed an informed consent form. Face-to-face interviews and one phone interview took place at a time and place of the participant’s choosing, with a duration between 45 and 60 minutes. Each interview was recorded (audio only), typed out *ad verbatim* and emailed to the participants for confirmation. The questions addressed important aspects for eHealth and is designed to inform the development of an eHealth intervention (Supplemental Material). At the time of the interviews, the intervention was in its development phase and reliant on the outcomes of this interview study for further and final development. The intervention was unknown to the general population and therefore none of the participants were involved with SAFE prior to the interviews. They did receive some information on the initial ideas for developing an eHealth intervention for providing information and options for help and support. When applicable we asked about personal experiences with IPVA. Furthermore, participants filled out a General Characteristics Questionnaire (GCQ) on demographical data (e.g., age, educational level) and their experience with IPVA (Table 2).

**Table 2.** Demographic Data and IPVA Experiences From Study Participants.

Participant	Age	Occupation Sector	Educational Level	Children	IPVA Type**	Professional Help***
101—S-P	41	Paid employment (business)	Vocational education	Yes	1,2,3,4	a,b,c,d,e
102—S-P	33	Freelancer	Higher vocational education	Yes	1,2,3	a,b,d
103—P	44	Freelancer	University	Yes	n/a	n/a
104—S-P	48	Public sector*	Postdoctoral	Yes	1,2	c
105—P	38	Public sector*	University	Yes	n/a	n/a
106—P	47	Freelancer	University	No	n/a	n/a
107—P	52	Public sector*	University	Yes	n/a	n/a
108—S-P	47	Public sector*	Secondary school	Yes	1,2,3,4	b,c,d
201—S	50	n/a	Higher vocational education	Yes	1,2,3,4	b,d
202—S-P	52	Public sector*	University	Yes	1,2	a,c,e
203—S	48	n/a	Higher vocational education	Yes	2	a,b,c,d
204—S	22	n/a	Vocational education	No	1	b,e
205—S	46	n/a	Vocational education	Yes	1	a,b,e
206—S	34	n/a	Vocational education	Yes	1,2	a,b,e
207—S	48	n/a	University	No	1,2,3,4	c
208—S	51	n/a	Vocational education	Yes	2	a,b,e

*Note.* P = professional, S = survivor, S-P = survivor-professional; *E.g., police, health care, education; **1 = physical, 2 = psychological, 3 = sexual, 4 = economic; ***a = GP practice (including psychological support), b = psychologist and psychiatrist, c = social worker, d = relationship therapist, e = DV/IPVA organization.

### Recruitment and Study Population

The survivors were contacted online through an organization that supports survivors of IPVA. The professionals were contacted online through DV and IPVA organizations. We used theoretical sampling. Participants were included if they were between 18 and 55 years old and self-identified as a survivor of IPVA and/or an expert on DV/IPVA. Exclusion criteria were: in need of immediate help and/or not speaking Dutch. We worked toward code and meaning saturation with a total of 16 women with various experiences of IPVA and an age range of 22 to 52 years old (Hennink et al., 2017). Originally, they were divided in two groups: 8 survivors and 8 professionals. However, while analyzing the data a third group was identified based on the unique input that they offered: survivor-professionals. These are professionals who have personal survivor experience, or survivors who have had training in using their own personal experiences to help others in dealing with IPVA. This group was then analyzed separately as their perspectives are shaped and blended by their experiences.

### Analysis

During the analysis of the interview data, we identified three groups based on the unique input they offered: survivors, professionals, and survivor-professionals. In comparing the input of these groups for an eHealth intervention, we expect the groups to provide diverse insights and, after identifying three groups instead of two, we expect the survivor-professionals to deliver the most input as they have a combined knowledge and experience from both perspectives. The final groups are defined as follows:

– Survivors: women who have experienced IPVA but who are not working on DV or IPVA as a professional, nor have they had any training to use their own experience in helping other people facing DV or IPVA (*N* = 7).
– Professionals: individuals who work on DV or IPVA as a professional, without personal experience of IPVA (*N* = 4).
– Survivor-professionals: professionals on DV and/or IPVA who have personal survivor experience, or survivors who have obtained specific training to use their personal experience to help others facing DV or IPVA (*N* = 5).

In analyzing the data we used the grounded theory approach (Henning et al., 2004). This approach uses qualitative content analysis, with open thematic coding, as a way of analyzing qualitative data for developing a theory. The grounded theory approach is based on inductive reasoning: “… the discovery of theory from data”—(Glaser & Strauss, 2017, p. 1). Raw data is shaped into codes, which are shaped into categories, which are then shaped into themes (Glaser & Strauss, 2017; Henning et al., 2004).

Two researchers (NvG, JtE) analyzed the interviews independently, employing an open thematic coding approach (Ayres, 2014; Glaser & Strauss, 2017; Henning et al., 2004). The qualitative data analysis program Atlas.ti, version 6.2 (Friese, 2011), was used to underline and code key terms. All personal identifiers were removed to avoid direct attribution of the illustrative quotes. Next, the researchers compared codes and coded segments and sought consensus about the coding frame during several iterations. After saturation was reached, all interviews were read again with the final codebook to check if all text segments had been coded correctly. To minimize loss of relevant information, a third researcher (KvRN) analyzed the first four interviews. The resulting codebook was organized into categories and themes by both researchers independently until consensus was reached again. All interviews were reread again to make sure that all data had been included.

## Results

In total, 16 women were interviewed between the ages of 22 and 52. All women were born in the Netherlands and identified as Dutch, living in four different provinces. All but one reported to be heterosexual (participant 203 answered “rather not say”). Regarding religious backgrounds: 9 answered “none/atheism,” 5 are Christian, and 2 answered “other.” In total, 10 out of 12 women who experienced IPVA reported that emergency services had been involved. Table 2 shows the participants’ demographic background and their experiences with IPVA.

**Figure 1.**
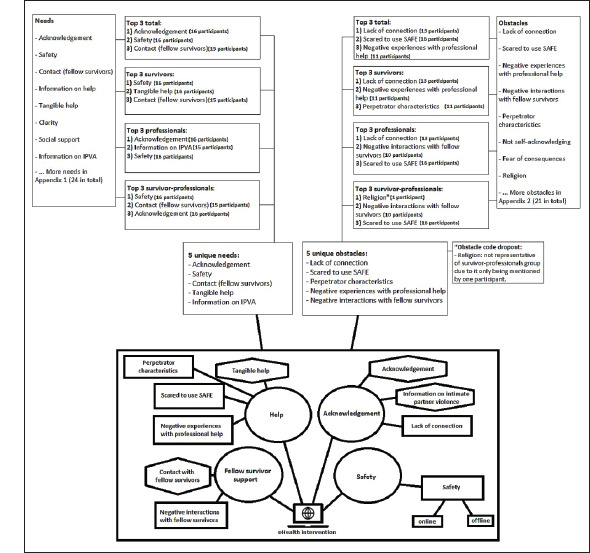
Main themes, categories, and codes.

*Note.* Circles are themes; rectangles are codes from the obstacle category; hexagons are codes from the needs category.

Codes were identified as needs (24 codes) and obstacles (21 codes). The most discussed codes were selected, looking at the top three mentioned codes (for needs and obstacles) for separate groups and in total (Appendices A and B). We made an in-depth assessment of the contents of ten codes in total (5 needs and 5 obstacles), showing similarities and differences between the three groups. The obstacle “religion” is discussed separately as it was mentioned by only one participant (survivor-professional). From the content analysis we extracted four overarching themes are as follows: safety, help, fellow survivor support, and acknowledgment (Figure 1). An overview of the feedback for the SAFE intervention itself can be found in Appendix C.

### Safety

#### Survivors.

Survivors discuss various aspects and contexts of safety. They want to be safe and feel safe, individually and with their children. They wish for (a) a safe space to live, (b) to know where they can go immediately if they are not safe; (c) protection during and after disclosing IPVA, and (d) help in reporting IPVA, in pressing charges, and leaving the partner. They discuss the fear of the partner finding out that they are looking for help.

204: “When something has happened and I want to leave or get help, you should tell me where to go and what to do. And that I would then indeed, preferably within 5 minutes, receive an answer with the possible options, such as I can go there or there right now, if it’s really unsafe for me.”

With regard to an online intervention they want to have a safe environment with independent advice from people who have sufficient expertise. They need to know that the information they share is not forwarded to (government) organizations without their consent.

206: “Many women are actually scared of the consequences of doing anything official or going to an official agency. It must be clear that it remains confidential and that there won’t be any notification. It should not be the case that if you’re looking for help that someone will think ‘oh, that is very serious, they really need help, I’ll arrange that’ (behind their back). That it remains in their own hands.”

It has to be clear that the intervention can be trusted. Being able to use it anonymously is important and it might also be safer since it can offer support without having to leave the house. A mobile phone is considered a safe device to use for such an intervention as you can always carry it with you and it can be locked.

#### Professionals.

Professionals mention the need for a safe environment where women are welcomed without being judged. In order to create safety social isolation should be decreased, e.g., by stimulating women to share their story and by informing DV professionals about the existence of SAFE.

103: “I’ve noticed that in such a situation they are completely isolated from the outside world. They don’t have any friends and only have sporadic contact with their family. To prevent relapse, I would raise publicity for this [SAFE] in the shelters, children’s health clinics, and also in women’s organizations.”

Professionals insist that the usage of an eHealth intervention depends partially on the women’s circumstances. If the woman´s life does not have a basic structure and routine, she will not be able to fit the internet-based offer into her life.

#### Survivor-professionals.

Survivor-professionals specifically mention concerns of personal safety, safety of the children and dependents and identification of trustworthy supporters. Survivors can struggle with (feelings of) unsafety after leaving the abusive partner.

101: “With the knowledge I have now, I’d be thinking about how to get out safely, with my children. Who believes me, who can I trust? Because it can be hard when Child Protective Services [CPS; in Dutch: Jeugdzorg] argues the child has the right to remain contact with the father. You’re totally burnt out, terrified of your ex, and you think ‘my children shouldn’t go there’, but they will. It drives you insane.”

Survivor-professionals confirm the need for a safe online environment, without judgment, recognizable for women in various IPVA situations and ideally accessible by mobile phone. Like the professionals, they mention the influence of personal circumstances on (online) help seeking behaviors.

202: “For someone to feel safe enough to participate they need some peace and quiet, a safe environment. Also mentally, you have to be open to it. I think the prerequisites are acknowledging that you have a problem and some level of safety.”

### Acknowledgment

#### Survivors.

Survivors say that the abuse, especially psychological and emotional abuse, gradually creeps in. It is not until later or after leaving the abusive relationship that they realize what happened.

201: “He made me totally dependent on him. I’ve stood on my own two feet ever since I was 17 years old, paid for my tuition and worked two jobs. I was never dependent on a man. But it creeped in. So bizarre. I could only see it in hindsight, due to therapy.”

Acknowledgment from others is important. This happens through contact with other women and survivor-professionals who have experienced IPVA, who understand and offer support. Acknowledgment can, furthermore, come from professionals that identify and verbalize the abuse taking place and that support accordingly.

Survivors say that acknowledgment and validation led them to be aware of their own situation. It helped them in gaining clarity and in reflecting, for instance on the unhealthy relationship dynamic and red flags. Acknowledgment and awareness are necessary to leave the violent situation and seek help. Furthermore, it is a sign that they are not alone in their experience with IPVA.

201: “Survivor-professionals and fellow survivors have been really helpful. Normal people who’ve also dealt with such an idiot. Acknowledgment, recognition, it’s very important. If I’d had an app like SAFE at the time, maybe I would’ve left him after a week of living together.”

#### Professionals.

Recognizing IPVA is not as easy as people may think, say the professionals. However, identifying it is very important as it is the first step toward help and many women do not recognize it for prolonged periods of time. This can be related to stereotypical images of IPVA, e.g., as a solely physical phenomenon, that are not applicable for many women.

103: “But you only start searching when you recognize it. And in my experience, 9 out of 10 times they only recognize it when it’s almost too late.”

105: “I also think of nuance. So not the traditional ‘dominant, dangerous man abuses defenseless, pathetic woman’. That’s still kind of the image people have of domestic violence but it’s not congruent with reality. I suspect many women do not identify with that image. And I think many women want the violence to stop but they don’t want the relationship to end. So nuance is important.”

Professionals say that the message that needs to be conveyed is: what IPVA is; that violence and abuse are not acceptable; that they are not the only ones experiencing it; that it happens at all levels of society; and that they should not be ashamed of it. Professionals talk about recognizing and respecting someone’s emotional process but they stress avoiding a perception of victimhood. Survivors should be empowered.

103: “I’d address women in an empowering manner. You are beautiful as you are and no one is allowed to hurt you. No one has the right. That they really understand this is bad for them. Address them in an empowering way, like ‘every woman is powerful’.”

They underline the need to speak to different women experiencing different forms of abuse to facilitate identification.

105: “Look, regarding partner violence women say: No, that’s not what’s happening here. But if you were to ask ‘Do you sometimes feel unsafe in your relationship?’ more women acknowledge that. It’s important to emphasize that you don’t have to be beaten up on a structural basis. It can also be about not being allowed to freely express your opinion.”

#### Survivor-professionals.

Survivor-professionals say that looking back they knew something was wrong but at the time they could not recognize it. They did not have that knowledge on IPVA, sometimes thought there was something wrong with themselves, they hoped it would not happen again, and did not know if and where they should look for help.

202: “I knew about it, in theory. I knew of women whose passports had been taken from them and were not allowed to do anything, who were not allowed to leave their house. I knew that those women are survivors. But I thought, I work, I have everything under control. I thought: I’m not a victim.”

102: “I didn’t know any of this, not the pattern, not the behaviors. I thought I was difficult. So you don’t look for help because you don’t know what you’re looking for.”

Acknowledgment is the first step in disclosing IPVA and seeking help. Ultimately, women need to acknowledge their situation to realize their own options for changing it. The role of professionals is to recognize and understand survivors’ needs and wishes and their dilemmas in leaving their partner.

108: “A professional is more eager to say you should leave. While the first steps are making sense of it in your head and sharing your story. I think the website shouldn’t propagate leaving. They will get defensive or resist if they feel pressured. And if they then still decide to stay, I think they should be able to find options about what they can do to make their situation more bearable.”

### Fellow Survivor Support

#### Survivors.

Survivors experienced more understanding from fellow survivors and survivor-professionals compared to people who have not experienced IPVA. Survivor networks provide support, opportunity to share and the intrinsic knowledge that your counterpart can empathize. The survivors suggest a chat and a buddy system for support and advice.

201: “I would’ve found it very helpful if someone had been there, a woman, who just understands and explains the steps [how to find a lawyer, housing etc.]. It’s in the app but you still have to make the call. Except I can’t call, I’m just crying. You need someone who believes you and picks up the phone for you.”

208: “If something’s on your mind, you can share it with the group. You receive reactions from people who’ve experienced it. Because the family doctor and professionals really don’t get it and you can never immediately get an appointment. In a Facebook or Whatsapp group you sometimes get a response within a minute, and that’s just great.”

However, contact with fellow survivors can be a negative experience as well: the stories can be retraumatizing; there can be mismatches in experiences or beliefs; and support or advice are sometimes negatively criticized by other survivors.

203: “Some people sink their teeth into it, they don’t recover, they get stuck. They will take on the victim role, cause friction, anger, and even depression. They really get depressed because they spend too much time in those Facebook groups.”

#### Professionals.

Professionals also find this type of contact important for survivors. It helps with disclosing and acknowledging IPVA, as well as with breaking taboos and encouraging action.

103: “Imagine this, I’m past the threshold of denial and I’m with a professional, but they say I’m part of the problem. I’ve noticed that women don’t like that, stuff like ‘you could’ve left’ or ‘how could you let this happen?’ You know, that type of judgment. That’s not what they need and maybe survivor-professionals are more empathic and understanding in those situations.”

The professionals state that negative interactions can occur with this type of contact: stories can trigger negative emotions and loss of hope; development of unhealthy friendships; and comparison of suffering (minimizing other people’s experiences). Furthermore, professionals urge that this contact should not limit women to a survivor role.

103: “Yes, there’s often a need for it. But what I do notice is that they search for the superlative degree. ‘You also experienced that? Yes but I... You had one black eye? Well, I had two’. You know like that, so they reinforce each other.”

107: “You could encounter an undesirable dynamic. Often times it’s people who have no social network. I felt that especially people with psychological problems were overrepresented somewhat, which can really leave its mark. For example, someone in a chat saying ‘no one listens to me, well then I’ll just go cut myself’. How do you respond to something like that?”

#### Survivor-professionals.

Survivor-professionals agree with survivors and professionals on the importance of contact with fellow survivors. It prevents or decreases social isolation, shame, and loneliness. This type of contact can be empowering and motivating to take action.

101: “Survivors understand that you can act crazy. They understand why, because of trauma, and they don’t judge. People who’ve never experienced it don’t really know anything. They think ‘stop being nervous, why are you acting weird’, you know.”

Survivor-professionals also acknowledge survivors can place excessive focus on their respective negative experiences. They state it may sometimes be better to speak with a survivor-professional instead of a fellow survivor, as survivor-professionals received training to prevent these negative interactions.

102: “The advantage of talking to a survivor-professional instead of to a survivor is that you don’t become completely mixed up in each other’s stories. You shouldn’t dwell on negative aspects of your experience. That tends to happen with survivors if they constantly talk to about the negatives. You should work toward processing it.”

### Help

#### Survivors.

Survivors express a need for tangible help that is congruent with their needs. Women need to know the exact steps they have or can take to leave the violent situation and/or to get help. These have to be explained in short and simple nonpressuring language. They want an overview of tangible help options.

201: “I always told my psychologist I need a step-by-step guide. Like, say I have to paint a strip. What do you need to do first, you have to sand it. Okay, sand it first. Then you need to clean it, remove dust. Then degrease it and then prime it. Those kinds of steps.”

Furthermore, it is important to consider practical help as well. Women do not only need legal and psychological help, they also need: shelters, help regarding finances, jobs and housing, and tips about stress relief. Survivors also mention buddies (survivors [-professionals]) as possibly helpful to support women in navigating help options and utilizing them.

207: “I’d hidden my credit cards etcetera in my neighbors closet. You know, they take everything away from you to isolate you. But he couldn’t get to those things, because I’d hidden them. So you need practical tips, for example about arranging things with the bank. Many women have no clue about this.”

Help from someone who understands what they have been through, for example a survivor-professional, is preferred. Official DV services and youth services are frequently not trusted by survivors. Some have negative experiences with professional help. For example, they speak of a lack of expertise and understanding, not being taken seriously, professionals telling them they cannot do anything to help (unless they agree to certain conditions), waiting lists, and a lack of a good fit between help and the woman’s needs.

205: “I didn’t want to press charges. I was scared, I knew he had a lot of connections. So hiding in the Netherlands with the kids didn’t feel safe. I wasn’t allowed to go abroad on account that my kids were under 12 years of age. The police was quick to say ‘We’ve offered you this, you can press charges. We can’t help you if you’re being difficult’.”

#### Professionals

Access to tangible help is important and survivors need to know where they can find it. Women should be approached with simple, nonpressuring, nonjudgmental information and help options. It should stimulate women to think about what they want and be applicable to various situations of IPVA and needs of survivors. However, one expert acknowledges that help from survivor-professionals could be a better fit with the woman’s initial needs, as they tend to be more empathic and understanding and give survivors time to tell their story. Professionals tend to take action and provide help and advice quickly, which might not correspond to the woman’s needs. They also point out that some professionals can be judgmental or pressure women toward leaving a violent partner. This can lead to negative experiences with professional help.

105: “Women don’t have to put up with it but they also don’t have to leave straight away. Ask them what it is they want. ‘You can work things out together’ is a very different message than ‘run for your life’.”

Some professionals use eHealth for their own clients, as an addition to face-to-face sessions. It can improve efficiency and optimize the necessary time commitment of both professional and client. Clients have the option to observe and reflect on certain things themselves and subsequently discuss it with the professional.

107: “For us, it [online help] is additional to the offline help. There are parts that people can do themselves that you hardly need to follow-up on. As long as it doesn’t touch upon the emotion or trauma, they can of course do many parts themselves.”

#### Survivor-professionals.

Similar to the survivors and professionals, survivor-professionals stress the importance of knowing which steps need to be taken to leave a violent situation; help options; and a sensitive, understanding approach. Survivor-professionals (for example buddies) could help in guiding women in these situations as it can be overwhelming and it involves uncertainty and grief. They agree on the notion that their group tends to be more empathic and patient, and less action oriented than professionals.

102: “At the beginning you can’t see the wood for the trees. So you have to provide guidance, a step by step plan. Put survivor-professionals in the database, they know what it entails. That really is a must in my opinion. Even good psychologists encounter problems with it if they have no personal experience [with IPVA].”

Survivor-professionals add that attention should be paid to various life dimensions (e.g., personal, relationships, health) as well. Women need this, but professionals do not always assess these needs.

202: “Those life domains, that’s good. I just had one issue: my ex. But often times others struggle with illiteracy, financial problems, relationship problems, you know. It all comes together. In the shelter they try to empower women at all levels to get them to be self-sufficient, like housing, finances, raising the children. Financial problems can evoke stress which may turn into violence. It is all intertwined.”

Furthermore, some survivor-professionals had negative experiences with professionals with regard to judgment and a lack of expertise and understanding. One survivor-professional argues that it is essential that the professional and the survivor analyze the IPVA situation (including perpetrator characteristics) in order to tailor the help to the woman’s specific context.

202: “If you think you’re dealing with domestic violence, you have to analyze the situation first. Because a narcissistic perpetrator isn’t the same as mutual violence. That really is far more complex. If you know nothing about this then… Like me and the professionals involved, we completely misjudged who we were dealing with.”

One survivor-professional declares that partly because of her partner being diagnosed with borderline and a personality disorder professional help advised her not to leave him.

101: “I went to an organization with a Christian background for psychological help. They said I couldn’t divorce him. Because it’s a sin, of course, but also because if I left him it would break his safe space, and he couldn’t handle that. So I waited for a while longer before I finally left.”

### Deepening the Results

Variations in perceived importance and priority of needs and obstacles between the three groups became visible while assessing the content and how many times a certain concept was mentioned during the interviews. Some needs and obstacles are mentioned by only one or two groups, which makes for interesting nuances in perspectives.

#### Survivors and survivor-professionals.

Survivors and survivor-professionals both mention trust, however professionals do not. Trust was discussed in the context of their social network, in professionals, and in using SAFE.

101: “When my husband and I went to the family doctor, he was very charming and interesting. And I was being extremely nervous, so guess who was the problem. The family doctor didn’t help me at all and so you lose confidence in the health care system. You don’t go there anymore. I didn’t talk to people around me either because my social network consisted of religious people telling me I wasn’t allowed to divorce him.”

They also bring up various obstacles in seeking help that professionals do not mention: children, practical problems, and loyalty or love.

108: “Eventually, I noticed that it was affecting my children. They were anxious too. I didn’t want to co-parent because I thought it wasn’t good for them. So I ended up going back to him because of the children. At the time I didn’t know about hiring a lawyer. When I closed the door behind me, my son said: ‘Mom, that’s the stupidest thing you have ever done.’”

202: “I struggled with it for a long time. Because you want him to have a role in the children’s upbringing. You don’t want to turn him in, he’s their father after all.”

#### Professionals and survivor-professionals.

Professionals and survivor-professionals both mention not wanting to be treated as a victim as a barrier to seeking help, this was not mentioned by survivors.

101: “Harsh terminology can shock you, we use the terminology: victims of domestic violence. That’s quite a harsh approach, you don’t want to be a victim of domestic violence. So you have to get used to that before you… although, you do want to present a clear image of who you are.”

Misinformation is also mentioned by both these groups as an obstacle. For example regarding expectations of professional help or as a control tactic by an abusive partner.

107: “What happens if I press charges? It means you request the police to prosecute someone. But it doesn’t mean that will actually happen. Especially when it involves domestic and sexual violence, there’s enormous friction for those women. Explaining this well helps. You shouldn’t discourage them but you have to be honest.”

#### Survivor-professionals.

Only survivor-professionals mention religion and malfunctioning electronics as obstacles.

However, religion was only discussed in one interview.

101: “I would add something on harmful traditional practices for people who are very religious. There are a lot of Christian people who are hard to reach. Women won’t leave, they can’t. They stay there till they die.”

#### Professionals.

Out of all the interview participants, only one professional mentions privacy legislation.

107: “I think many people like something like Whatsapp. Of course you have to navigate the GDPR (AVG in Dutch), which is very difficult.”

#### SAFE specific feedback.

Furthermore, all groups proposed feedback specifically for the SAFE intervention (Appendix C). The groups mention 11 similar points of feedback, but each group also points out unique features. Professionals offer 3 unique points of feedback, while survivors and survivor-professionals each offer 10 unique points of feedback. For example, only professionals talk about how the intervention being completely online is safer than having to put things on paper. Survivors are the only ones who talk about how contact must feel like real contact, not like talking to a robot. And only survivor-professionals mention how it is important to include various life domains, such as finances and housing, in the intervention.

## Discussion

This is the first study to examine not only the needs, obstacles and wishes of survivors with regard to eHealth for IPVA but to also include professionals’ insights and the unique perspective from a hybrid type of involved party: survivor-professionals. Our results show that these three groups are largely congruent in their feedback on what women in IPVA situations need and what obstacles they face. However, there are differences between the groups, showing that the survivor-professionals are a separate category next to survivors and professionals that should not be excluded from target group-oriented participatory research.

All groups largely agree on the importance of safety, acknowledgment, contact with fellow survivors, and help. They mention the importance of tangible help options, acknowledgment, and an approach that is sensitive to various experiences of IPVA, with matching information and help options. Furthermore, they state that providing contact options with survivors and (survivor-)professionals is vital. With regard to technical aspects they talk about safety measures, anonymity, and easy access. This is consistent with findings from qualitative studies on online help and IPVA or dating violence in Australia (Tarzia et al., 2018, 2017) and the United States (Lindsay et al., 2013): women view an app or website as an appropriate way to seek help in IPVA situations, with the appealing possibility of 24/7 easy access and anonymity.

However, the groups appear to differ in their prioritization of needs and obstacles. When describing needs, for survivor-professionals and survivors the primary discussion topic was safety, while for professionals it was acknowledgment. This might be explained by differences in personal involvement in IPVA. Survivors and survivor-professionals share a personal experience of IPVA, which may highlight their prioritization of feeling and being safe (ten Boom & Kuijpers, 2012). Professionals on the other hand want to help women to self-acknowledge their situation and design an online intervention that survivors can identify with. This appears as a result- and solution-oriented approach, which complies with claims from the interview data that professionals are often quick to take action and provide advice, their training in actively helping people, and the belief that the first step of help seeking is acknowledging the situation you are in, as professionals have learned from applying the Stages of Change model (Frasier et al., 2001; Zink et al., 2004). With regard to safety and help, survivor-professionals and professionals insist there must be some safety and peace to use an eHealth intervention. Survivors on the other hand, express a need for acute help. Since an eHealth intervention may not always be suitable to that need, it is important to manage survivors’ expectations and provide options for acute help.

A unique aspect mentioned solely by the survivor-professionals was the focus on various life domains, such as financial situation, housing, and child rearing. These components are often overlooked in online interventions for women exposed to IPVA but are very important (Rempel et al., 2019). Rempel et al. (2019) state that existing online interventions mostly focus on safety planning. While safety is crucial, they stress that women also need support with regard to housing, finances, child care etc. to help them move from the abusive relationship and sustain their independence.

With regard to obstacles, both professionals and survivors discussed a lack of connection the most. For the survivors, this might have been a serious obstacle in their own experience with IPVA and help seeking. For the professionals, connection is important but from their own perspective, which means that it can represent an obstacle they face themselves when trying to build rapport. Connection difficulties might be a mutually experienced phenomenon due to the emotional difficulty, stigma, frequent denial, and the long process of gradual acknowledgment and change. For example, survivors may feel victim blamed by professionals (Crowe & Murray, 2015), and professionals may struggle with their own hesitations and biased perceptions regarding IPVA, such as whose responsibility it is to intervene and who is perceived as a “credible” survivor (Robinson, 2010; Virkki et al., 2015). Both may experience differences in understanding the situation and necessary actions, for example in risk assessment (Cattaneo, 2007), which can make for a challenging interaction and help process.

Although all groups see the benefits of (survivor) support networks, for example in increasing acknowledgment and safety and decreasing social isolation, especially the survivor-professionals highlighted the importance of negative interactions with fellow survivors. This may relate to complicated previous experience with fellow survivors, as negative social reactions can have an adverse impact on survivors’ mental health (Sylaska & Edwards, 2014). This stance by professional-survivors highlights the professional distance and skills they acquired through their training and their added value as a bridge between professionals and survivors (Storms et al., 2020; Van der Kooij & Keuzenkamp, 2018).

Structurally, the survivor-professionals emerged as a hybrid and yet unique group, combining personal experience and expert knowledge. Investigating them highlighted the nuances in priorities and perspectives between the different stakeholders involved in the support of IPVA survivors. Considering these differences is essential in developing a tool that can serve both survivors and the community that supports them. As an eHealth intervention takes on a (professional) supportive role, while still allowing survivors to share their experiences with each other without external guidance, it will have to reproduce some of these nuances. The eHealth platform might in itself represent a hybrid function (Kreps & Neuhauser, 2010; Kuenne & Agarwal, 2015; Vollert et al., 2019), thus, the acknowledgment and careful consideration of this input is essential.

### Strengths and Limitations

A strength of this study lies in the sample consisting of three groups, providing unique perspectives. Furthermore, the sample includes participants with a variety of experiences with IPVA and (professional) help, and a variety of participating organizations from various regions in the Netherlands. The selected sample covers a broad age range and variety in educational level, as well as women with and without children.

Limitations of this study include a lack of variation in cultural and religious backgrounds, as many participants are white and atheist or Christian. Moreover, the sample largely consisted of heterosexual women. They all seemed to have easy access to the internet and at least a basic level of digital literacy. This could be different for certain (small) groups in the Netherlands, although the vast majority of the Dutch population has access to the internet and is digitally literate, resulting in potential limitations in access that stay hidden in this study. Currently, we are conducting a study with women with migrant backgrounds to explore these issues. As we know IPVA occurs in all layers of society and in various types of relationships, a more diverse sample is recommended in future studies. Furthermore, the contents of the interviews disclosed the survivor or professional status of participants and this might potentially sharpen differences between these groups, which we would not necessarily have found without this disclosure. In developing SAFE, attention should be paid to diversity on various levels to match with various groups of women who experience IPVA. We should also acknowledge men as survivors of IPVA and their diverse backgrounds and experiences. They could possibly benefit from an eHealth intervention as well.

### Implications

Online interventions for women experiencing IPVA should be developed in a participatory manner involving individuals, who have diverse firsthand experience. Survivors, survivor-professionals, and professionals are groups that offer valuable real-world insights essential for enhancing help options for IPVA survivors and in creating a successful eHealth intervention. In (professionally) supporting IPVA survivors, we need to acknowledge that perspectives from survivors, professionals and survivor-professionals can differ. For example, professionals should be aware of the survivors’ need for direct, practical help and with regard to support from fellow survivors, we need to consider that this support could be more helpful for survivors when it is monitored on a platform by people who (also) have professional knowledge on IPVA. The results from this interview study were used in the development of the SAFE intervention (van Gelder et al., 2020). For the aforementioned examples this means that we have included community managers with professional knowledge on IPVA that monitor the interactions between survivors and who can intervene when necessary. Also, it means SAFE includes help options that offer the direct, practical help that survivors are looking for and, for safety, an escape button that immediately closes the intervention and shows a neutral website. Future research includes quantitative and qualitative evaluations with survivors who use the SAFE the intervention. Our study supports the need for an eHealth intervention like SAFE and the importance of involving diverse groups in developing interventions, including hybrid groups like survivor-professionals, which blend different experiences.

## Appendices

## Appendix A. Needs.

**Table A1.** Overview of all Codes in Needs, Ranking Per Group and in Total.

Need	Survivors	Professionals	Survivor-Professionals	Total
Acknowledgment	47 *(3)*	48 *(1)*	43 *(3)*	138
Safety	66 *(1)*	19 *(3)*	49 *(1)*	134
Contact (fellow survivors)	44 *(4)*	9 *(12)*	46 *(2)*	99
Information on help	42 *(5)*	18 *(4)*	39 *(4)*	99
Tangible help	62 *(2)*	12 *(8)*	21 *(10)*	95
Clarity	41 *(6)*	14 *(6)*	28 *(7)*	83
Social support	40 *(8)*	11 *(10)*	32 *(6)*	83
Information on IPVA	20 *(12)*	22 *(2)*	37 *(5)*	79
Accessibility	41 *(7)*	14 *(7)*	23 *(9)*	78
Anonymity	26 *(9)*	11 *(11)*	18 *(12)*	55
Control	21 *(11)*	12 *(9)*	20 *(11)*	53
Need for SAFE	16 *(14)*	9 *(13)*	15 *(14)*	40
Awareness	7 *(18)*	18 *(5)*	14 *(15)*	39
No judgment	3 *(21)*	6 *(18)*	27 *(8)*	36
Language use	17 *(13)*	8 *(14)*	7 *(19)*	32
Trust	24 *(10)*	0	5 *(21)*	29
Telling your story	9 *(17)*	7 *(16)*	12 *(16)*	28
Understanding	13 *(15)*	5 *(19)*	10 *(17)*	28
Well-functioning website	0	8 *(15)*	17 *(13)*	25
Expertise	13 *(16)*	2 *(20)*	6 *(20)*	21
Not being treated as a victim	0	7 *(17)*	9 *(18)*	16
Direct contact	7 *(19)*	1 *(21)*	2 *(22)*	10
Hope	5 *(20)*	1 *(22)*	1 *(23)*	7
Warm and inviting	2 *(22)*	0	0	2
Total	566	262	481	1,309

*Note.* The cursive numbers between brackets represent the quantitative ranking of the needs per group.

## Appendix B. Obstacles.

**Table B2.** Overview of all Codes in Obstacles, Ranking Per Group and in Total.

Obstacle	Survivors	Professionals	Survivor-Professionals	Total
Lack of connection	28 *(1)*	15 *(1)*	9 *(6)*	52
Scared to use SAFE/ehealth	18 *(4)*	14 *(3)*	15 *(3)*	47
Negative experiences with professional help	22 *(2)*	8 *(5)*	15 *(4)*	45
Negative interaction with fellow survivors	12 *(6)*	15 *(2)*	17 *(2)*	44
Perpetrator characteristics	20 *(3)*	5 *(7)*	13 *(5)*	38
Not self-acknowledging	10 *(7)*	14 *(4)*	7 *(8)*	31
Fear of consequences	17 *(5)*	2 *(9)*	5 *(11)*	24
Religion	0	0	23 *(1)*	23
Lack of social support/isolation	9 *(8)*	1 *(11)*	9 *(7)*	19
Shame	6 *(10)*	6 *(6)*	4 *(13)*	16
(Suitable) Help not available	9 *(9)*	2 *(10)*	1 *(14)*	12
Children	5 *(13)*	0	7 *(9)*	12
Practical	6 *(11)*	0	5 *(12)*	11
Guilt	2 *(16)*	1 *(12)*	6 *(10)*	9
Distrust	6 *(12)*	1 *(13)*	1 *(15)*	8
Lack of privacy	3 *(14)*	3 *(8)*	1 *(16)*	7
Loyalty/love	3 *(15)*	0	1 *(17)*	4
Misinformation	0	1 *(14)*	1 *(18)*	2
Privacy legislation	0	1 *(15)*	0	1
Malfunctioning electronics	0	0	1 *(19)*	1
Gloom/giving up	1 *(17)*	0	0	1
Total	177	88	141	406

*Note.* The cursive numbers between brackets represent the quantitative ranking of the obstacles per group.

## Appendix C. Specific Feedback for SAFE Intervention.

**Table C3.** Needs, Obstacles, and Feedback Specifically for SAFE Intervention.

Escape button: good for (feelings of) safety.	P ☑ S ☑ S-P ☑
Safe usage: explanation of how to delete browser history and use incognito mode.	P ☑ S ☑ S-P ☑
Safe means of communication with participants.	P ☑ S ☑ S-P ☑
Approach: women have to recognize themselves in the way SAFE approaches them, in the presentation of information and experiences from survivors. Discuss abuse, feeling unsafe or unsure, various types of violence and abuse. Avoid stereotypical, black and white messages.	P ☑ S ☑ S-P ☑
Diversity: consider diverse IPVA situations that women can identify with and cater to a variety of needs. E.g., a checklist, examples of behavior that portrays various types of IPVA.	P ☑ S ☑ S-P ☑
Interactive: a chat, forum, and short movies with survivors are good additions.	P ☑ S ☑ S-P ☑
Chat and forum: important to provide, it could be helpful for some women in sharing their story. Women should be able to use it anonymously. Make sure to monitor and quickly stop negative interactions, perhaps provide an ignore/report option. A chat is a fast and easy way of communicating. The chat could be available 24/7, as some survivors might want to chat during the night, even if you cannot monitor 24/7. However, be cautious with women getting dependent on it or focusing so much on helping others that they forget about themselves.	P ☑ S ☑ S-P ☑
Tangible (professional) help and tips: to refer to via a database. Provide an overview of tangible help options (including links to their websites, how to contact them, etc.) with filters for types of help and region. Include survivor-professionals in this overview. Also, provide information on what you can expect from certain types of help. Furthermore, provide tangible tips on practical issues, e.g., when a survivor’s preparing to leave the violent partner.	P ☑ S ☑ S-P ☑
Dissemination: disseminate the existence of SAFE so it is easy to find. E.g., through DV and mental health organizations, women’s organizations and the consultation bureau for infants and toddlers. Professionals should know SAFE as well in order to refer their patients/clients.	P ☑ S ☑ S-P ☑
Thresholds: for using SAFE may be not wanting to feel like a victim or not identifying with the image of IPVA. Another threshold can be the corresponding RCT study. It is important to give participants the option to be anonymous and to let them know what happens with personal information and data. Another threshold can be the fear of the (ex-)partner finding out that they use SAFE or encountering their (ex-)partner on the website.	P ☑ S ☑ S-P ☑
Feeling safe: to use the intervention depends on being able to use it anonymously, and on safety measures. E.g., an escape button and measures regarding browser history. It also depends on being able to stay in control (help is organized at their own initiative) and on perpetrators not being able to gain access.	P ☑ S ☑ S-P ☑
Publicity: SAFE has to be known in society as a help option when dealing with IPVA.	P ☑ S ☑ S-P ☑
Screening: of all registrations as a safety measure and to avoid (ex-)partners who try to infiltrate. Be aware of hacking.	P ☑ S ☑ S-P ☑
Emergency contact: only optional and participants decides when contact is permitted.	P ☑ S ☑ S-P ☑
Help options: should be broad so it connects with various needs that women may have. They have to be tangible and relevant for different types of IPVA situations. They should also entail options for contact with fellow survivors and survivor-professionals.	P ☑ S ☑ S-P ☑
Themed chats: with survivor-professionals and professionals are a good addition.	P ☑ S ☑ S-P ☑
Neutral appearance: it should not be clear directly that it is about DV or IPVA.	P ☑ S ☑ S-P ☑
Completely online: the registration process, providing information, etc., all takes place online. This is safer than putting it on paper, which is often the case in regular professional help.	P ☑ S ☑ S-P ☑
Problem solving skills: be careful with providing exercises, as it is not clear how this will be interpreted and put into practice, with possible negative consequences (violence escalating). Additional help options to guide women in using these tips and skills should be provided.	P ☑ S ☑ S-P ☑
Safety module: on being safe at home, which organizations can help you to be safe etc.	P ☑ S ☑ S-P ☑
Personal information from participants has to be protected.	P ☑ S ☑ S-P ☑
Digital diary: can be safer online than to have it lying around the house.	P ☑ S ☑ S-P ☑
Access: certain webpages should only be available for people who can log in.	P ☑ S ☑ S-P ☑
Awareness: educate women on what is (ab)normal and/or (un)healthy in relationships so they can recognize red flags in their own relationship.	P ☑ S ☑ S-P ☑
Children: educate women on the impact of IPVA on their children so they recognize IPVA is dangerous for their children as well.	P ☑ S ☑ S-P ☑
Free usage: participants should be free to use modules according to their own needs and at their own pace, not in a predetermined sequence.	P ☑ S ☑ S-P ☑
Real contact: it is important to have a sense of real contact, not as if you talk to a robot.	P ☑ S ☑ S-P ☑
Immediate danger: provide options for women who are in immediate danger.	P ☑ S ☑ S-P ☑
Contact: create safety, trust, and warmth in contact with survivors, to prevent drop out.	P ☑ S ☑ S-P ☑
Personal information: gather as minimal as possible and declare it is stored safely.	P ☑ S ☑ S-P ☑
Private messaging: possibly helpful when some does not want to communicate in a group.	P ☑ S ☑ S-P ☑
Safe appearance: background colors have to be inviting but should not be too bright with regard to brightness from the screen when looking at it at night and someone noticing it.	P ☑ S ☑ S-P ☑
Information: has to be recognizable and tangible in order to be aware of your own situation and what steps you can take to get out.	P ☑ S ☑ S-P ☑
Search option: perhaps provide this to search within the website easily.	P ☑ S ☑ S-P ☑
Information: on types of IPVA but also on types of perpetrators.	P ☑ S ☑ S-P ☑
Life domains: provide information, advice, and options for various life domains.	P ☑ S ☑ S-P ☑
Checklist: perhaps provide a test/checklist on the situation which, based on your answers, will then refer you to specific types of help and organizations.	P ☑ S ☑ S-P ☑
Logically structured: women have to know where they can start, how they can use SAFE, and where they can go if they want additional help or information.	P ☑ S ☑ S-P ☑
Safe space: it is important that SAFE is a safe, non-judgmental space for women to go through the process at their own pace.	P ☑ S ☑ S-P ☑

*Note.* P = professionals, S = survivors, S-P = survivor-professionals.

## Supplemental Material

Data are available upon reasonable request from the authors. Supplemental material (interview guide) for this article is available online. Click here for additional data file. Supplemental Material for “If I’d Had Something Like SAFE at the Time, Maybe I Would’ve Left Him Sooner.”—Essential Features of eHealth Interventions for Women Exposed to Intimate Partner Violence: A Qualitative Study by Nicole van Gelder, Suzanne Ligthart, Julia ten Elzen, Judith Prins, Karin van Rosmalen-Nooijens and Sabine Oertelt-Prigione, in Journal of Interpersonal Violence

## Author Biographies

**Nicole van Gelder**, MSc, is a PhD student at the Department of Gender in Primary and Transmural Care at the Radboudumc. She has a background in pedagogical sciences and her research focuses on intimate partner violence and abuse, and developing and evaluating eHealth for women exposed to this type of violence.

**Suzanne Ligthart**, MD, PhD, is a family physician in Nijmegen and researcher at the Department of Primary and Transmural Care of the Radboudumc, Nijmegen. Her research interests lie in mental health, communication, and mixed methods research.

**Julia ten Elzen**, BSc, is a medical student at the Radboud University Nijmegen. Prior to starting her clinical rotations, she decided to participate in the research on SAFE. She is mainly interested in the social and psychiatric aspects of medicine.

**Judith Prins**, PhD, is a clinical psychologist and full professor at the Department of Medical Psychology. Her research involves the development and testing of eHealth interventions for cancer survivors.

**Karin van Rosmalen-Nooijens**, MD, PhD, is a family physician and researcher at the Department of Primary and Transmural Care of the Radboudumc Nijmegen. Her areas of interest are eHealth, Violence against Women, Sexual Medicine and Family Violence in Primary Care. She currently works as a FP, researcher, and sexologist.

**Sabine Oertelt-Prigione**, MD, PhD, MSc, is professor of sex and gender-sensitive medicine (SGSM) at the Radboud University Medical Center. She focuses on the development of practice-oriented methods for SGSM and the institutionalization of the topic. She has extensive experience in the development of preventative measures for IPV, sexual harassment, and gender discrimination and is focusing on innovative solutions for their delivery.
