# Supplementary material for: “If I’d Had Something Like SAFE at the Time, Maybe I Would’ve Left Him Sooner.”—Essential Features of eHealth Interventions for Women Exposed to Intimate Partner Violence: A Qualitative Study
Source: J Interpers Violence. 2021 Aug 6;37(19-20):NP18341–75. doi: 10.1177/08862605211036108 (PMC9554282; doi:10.1177/08862605211036108)
Supplement: Data are available upon reasonable request from the authors. Supplemental material (interview guide) for this article is available online. [file sj-pdf-1-jiv-10.1177_08862605211036108.pdf]

Supplementary material: interview guide (main questions – translated to English).

The interviewer introduced herself and the project that the interview study is part of.

Questions were only asked when applicable since we interviewed various groups.

- Could you introduce yourself? (age, occupation, family etc.)
- If you have a personal experience of IPVA, could you tell me about the nature of this violence? / Could you tell me about the nature of the IPVA that you experienced?
- Are you currently experiencing IPVA?
- Do you have any experience with online help?
- Do you think there's a need for a website among women experiencing IPVA? (Would you feel the need for such a website when you experienced IPVA?)
- Imagine you would look for help online. What would be important for you?
- According to you, which devices and channels would you / women experiencing IPVA use to talk about what they (have) experience(d)?
- An important aspect of an online intervention is safety: what do you think are prerequisites for women to use an online intervention? / What would be needed to make you feel safe enough to use an online intervention?
- [Escape button was explained.] Do you think an escape button is needed and why (not)?
- Regarding design, what would you find important in the lay-out of a website?
- We are thinking of offering modules containing information on for example IPVA, healthy relationships, professional help, support, and problem-solving skills. What do you think of this?
- What type of information would you like to find in an online intervention?
- What do you think of adding short movie clips (with survivors and professionals) to the online intervention?

- Do you think women who experience(d) IPVA would be in need of a chat or forum on the website?
- Do you have any other tips or remarks you would like to share with us for the development of the online intervention?
- Do you have any further questions about the interview or the research project?
